# Associations between spinal flexibility and bracing outcomes in adolescent idiopathic scoliosis: a literature review

**DOI:** 10.1186/s13018-023-04430-z

**Published:** 2023-12-11

**Authors:** Chang Liang Luo, Christina Zong Hao Ma, Yi Ying Zou, Li Sha Zhang, Man Sang Wong

**Affiliations:** 1 Department of Biomedical Engineering, The Hong Kong Polytechnic University, Hung Hom, Kowloon, Hong Kong SAR; 2 Department of Prosthetic and Orthotic Engineering, School of Rehabilitation, Kunming Medical University, Kunming, China; 3 Suzhou Vocational Health College, Suzhou, China

**Keywords:** Adolescent idiopathic scoliosis, Spinal flexibility, Bracing, Predictor

## Abstract

**Objectives:**

To identify the existing assessment methods used to measure the spinal flexibility of adolescents with idiopathic scoliosis before bracing and to evaluate the predictive effect of spinal flexibility on bracing outcomes.

**Methods:**

A broad literature search was performed in the PubMed, Web of Science, EMBASE, CINAHL, Scopus, and Cochrane Library databases to obtain relevant information about spinal flexibility and bracing outcomes. All literature was retrieved by October 14, 2023. The inclusion and exclusion criteria were meticulously determined. The quality of each included study and the level of evidence were evaluated by the Quality in Prognosis Studies (QUIPS) method and the Grading of Recommendations, Assessment, Development, and Evaluation (GRADE) system, respectively.

**Results:**

After screening 1863 articles retrieved from databases, a total of 14 studies with 2261 subjects were eligible for the final analysis in this review. Overall, nine methods of flexibility assessment were identified, including supine radiographs, supine lateral bending radiographs, lateral bending radiographs but without clear positions, hanging radiographs, fulcrum bending physical method, and ultrasound imaging in the positions of supine, prone, sitting with side bending and prone with side bending. In addition, five studies demonstrated that flexibility had a strong correlation with in-brace correction, and eleven studies illustrated that spinal flexibility was a predictive factor of the bracing outcomes of initial in-brace Cobb angle, initial in-brace correction rate, curve progression, and curve regression. The results of GRADE demonstrated a moderate-evidence rating for the predictive value of spinal flexibility.

**Conclusion:**

Supine radiography was the most prevalent method for measuring spinal flexibility at the pre-brace stage. Spinal flexibility was strongly correlated with the in-brace Cobb angle or correction rate, and moderate evidence supported that spinal flexibility could predict bracing outcomes.

## Introduction

Adolescent idiopathic scoliosis (AIS) is an unexplained pathological deformity of the spine characterized by a coronal curvature of more than 10°, with axial rotation of the apex and sometimes with sagittal malalignment. Approximately 0.47–5.2% of teenagers aged 10–16 were diagnosed with AIS [1], especially girls. AIS may cause serious physical problems and mental issues, which are considered to be a heavy burden for patients and their families [2].

According to the latest version of the Society on Spinal Orthopedic and Rehabilitation Treatment (SOSORT) guidelines, the nonoperative treatment methods for AIS include observation, special inpatient rehabilitation (SIR), physiotherapeutic scoliosis-specific exercises (PSSE), and bracing [3]. High-quality studies confirmed the effect of bracing on preventing curve progression and even reducing it [4–6]. Different factors have been researched as predictors of bracing treatment outcomes. For instance, Sun et al. found that maturity, curve type, and curve size were independent risk factors for curve progression with bracing treatment [7]. In Steen’s retrospective study, good brace adjustment and compliance were proven to be the best predictors of long-term success [8]. Boggart et al. found that initial in-brace correction was a strong factor for predicting treatment failure, brace wear time was a moderate-evidence predictor, and original curve degree and type were not associated with brace treatment outcomes [9].

The coronal deformity angular ratio (C-DAR), calculated with the maximal Cobb angle divided by the number of vertebrae in the curve [10], was also determined as an independent predictor of long-term bracing outcome in Babaee et al.’s study [11].

In addition to these factors, spinal flexibility is also an important factor for planning the treatment of AIS, which is usually used to assist surgeons in defining the fusion strategy and predicting the surgical results [12]. The predictive value of spinal flexibility for brace correction outcomes has also received widespread attention but has not yet reached a unanimous conclusion. Clin et al. conducted a simulation study and found that the average quantity of immediate correction required to eliminate the bending moment was 48% for the flexible spine model and 27% for the rigid spine model, which suggested that brace treatment can be more efficient when spinal curves are more flexible [13]. Cheung et al. [14] and He et al. [15] observed significant associations between spinal flexibility and in-brace correction, while in recent studies, Falbo et al. [16] and Strube et al. [17] demonstrated no correlation between spinal flexibility and brace treatment success. It is thus far ambiguous whether spinal flexibility could estimate the effect of bracing, and the inconsistency of the findings makes it hard for clinicians to provide adequate prognostic information to patients. To this end, it is essential to identify, evaluate, and integrate all existing evidence relevant to spinal flexibility and its predictive effect on bracing outcomes to provide guidance for orthotists and patients.

In addition, various approaches have been utilized to assess spinal flexibility. In the review study of He et al., eleven kinds of radiographic assessment methods for spinal flexibility were identified [18]. Ultrasound imaging and magnetic resonance imaging (MRI) have also been used on surgical candidates to measure spinal flexibility [19, 20]. However, which method is more suitable for measuring spinal flexibility in bracing candidates has not been well identified. Therefore, this review aims to (1) identify the assessment methods used to measure spinal flexibility before the treatment of bracing and (2) evaluate the predictive effect of spinal flexibility on bracing outcomes to collate the updated evidence and provide recommendations for physicians and orthotists when making clinical decisions.

## Methods

### Search strategy

This review was conducted according to the guidelines of the Statement of Preferred Reporting Items in Systematic Reviews and Meta-Analyses (PRISMA), and the literature retrieval was performed on the PubMed, EMBASE, Web of Science, Scopus, CINAHL (Complete), and Cochrane Library databases to identify relevant studies. Google Scholar was manually searched to track the possibly useful articles from the reference lists of relevant studies not recognized by the electronic database searches.

Key search items include “adolescent idiopathic scoliosis,” “AIS” or “idiopathic scoliosis,” and “flexibility” or “rigidity” or “correctability” or “reducibility,” and “brace” or “bracing” or “orthotics” or “orthosis” or “orthoses” or “conservative treatment” or “nonsurgical treatment” or “nonoperative treatment.” The combination of these items, together with the Boolean operators “AND” and “OR,” varied with the retrieval engine. The detailed retrieval strategy in PubMed is presented in Table 1. The whole literature search process was completed before and on October 14, 2023.

**Table 1:** Retrieval strategy in PubMed

(“Adolescent idiopathic scoliosis” OR “AIS” OR “idiopathic scoliosis”)
AND
(“Flexibility” or “rigidity” or “correctability” or “reducibility”)
AND
(“Brace” or “bracing” or “orthotics” or “orthosis” or “orthoses” or “conservative treatment” or “nonsurgical treatment” or “nonoperative treatment”)
Filters used
Language: English

### Inclusion and exclusion criteria

The following criteria determined which studies could be included in this systematic review: (1) subjects were diagnosed with AIS; (2) subjects were treated with bracing; (3) flexibility was one of the indicators with a clear description of the measurement method; (4) studies described the bracing treatment outcomes; (5) studies analyzed the association between flexibility and bracing outcomes; and (6) full text was available. Any model or simulation study, case report, editorial, comment, letter, guideline, protocol, review article, and any literature written in a language other than English were excluded.

### Study selection

One reviewer (R1) searched the database and obtained the preliminary records for title and abstract screening. Two other reviewers (R2 and R3) independently evaluated article titles and abstracts for eligibility based on the above inclusion and exclusion criteria. They sorted the results of their screenings into distinct Microsoft Excel files according to the terms of inclusion, exclusion, and undefined. The articles that were sorted as inclusion and undefined were considered for full context review. Any uncertainty or disagreement about the final study selection was determined after discussing with the first reviewer (R1).

### Risk of bias assessment and level of evidence

Each included studies were subjected to a quality assessment with the modified Quality in Prognosis Studies (QUIPS) tool [21, 22], a critical appraisal instrument used to evaluate the quality of studies of prognostic factors. Six domains are considered during the assessment of the risk of bias: study participation, study attrition, study confounding, outcome measurement, prognostic factor measurement, and study analysis and reporting. Each domain is scored as 2, 1, or 0. Articles were identified as high quality when they scored 2 for all six domains, namely, the overall score was 12 [23]. If the overall score was 11, the study was determined to be of moderate quality. When a study was scored ≤ 10, it was defined as low quality [23]. Two reviewers (R2 and R3) assessed the methodological quality of the studies, and disagreements were resolved by consulting the first reviewer (R1).

Moreover, the level of evidence for the predictive factor was determined in accordance with the Grading of Recommendations, Assessment, Development, and Evaluation (GRADE) system by reviewers. The significance of the evidence level related to spinal flexibility can be rated as “high,” “moderate,” “low,” and “very low” [24]. The study design determined the level of evidence, but additional considerations may degrade and upgrade the certainty of evidence. Risk of bias, inconsistency, indirectness, imprecision, and publication bias are factors that downgraded the quality level of evidence, while moderate or large effect size, dose effect, and confounders may increase the evidence rating [24–26].

A kappa statistical analysis was conducted to test the consistency of the assessment results by the two reviewers [27].

### Data extraction

Data were extracted in the same way by the reviewers, who independently performed the study selection and quality assessment. Information regarding the first author and publication year, study type, population, sample size, age of subjects, initial Cobb angle, flexibility rate, measurement methods of flexibility, type of brace, duration of brace treatment and follow-up, bracing treatment outcomes, and study results were recorded. All studies included in this review were listed in a standardized data form. Basic information and data on spinal flexibility and treatment outcomes were documented in the results. The missing information in any study relevant to this review was collected by sending emails to the corresponding authors.

### Synthesis and analysis of results

The assessment techniques and corresponding positions of spinal flexibility were summarized according to the descriptive information of the included literature. The correlation of flexibility as a predictive factor with the bracing outcomes was displayed by the effect measures of the correlation coefficient (*r*) or the odds ratio (OR) with a 95% confidence interval (CI).

Meta-analysis was not performed in this systematic review due to the high degree of heterogeneity in the included studies for the various kinds of bracing and spinal flexibility measurement methods.

## Results

### Study inclusion

**Fig. 1:**
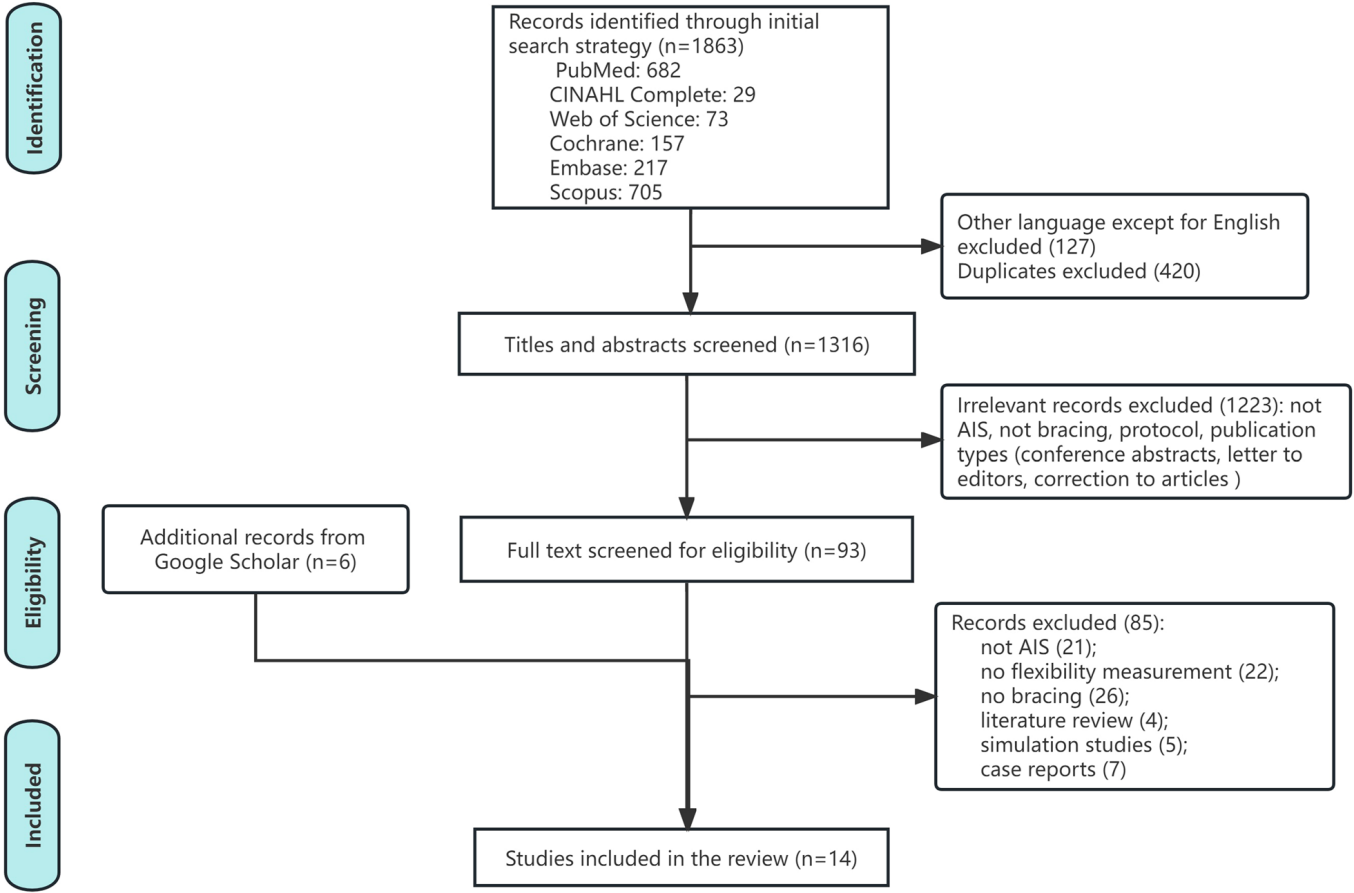
PRISMA flow diagram of literature screening

The initial search yielded 1863 potentially eligible publications from the six databases. After filtering the English-published literature and removing duplicate records with Endnote software (Endnote 20.4.1 for Windows, Clarivite ™, USA), a total of 1316 records remained for the screening of titles and abstracts. Based on the inclusion and exclusion criteria, 1223 records were further excluded, and the full texts of the remaining 93 studies were screened. Six additional studies were identified through backward citations from the reference lists of the eligible studies and were searched by Google Scholar. After reading the full context of 99 papers, a total of 14 studies were finally included in this systematic review. Figure 1 illustrates the screening process using a PRISMA flow diagram.

### Risk of bias of included studies and rating of evidence

Table 2 shows the results of the QUIPS appraisal. Of the 14 articles included, five (59.4%) had a low risk of bias [6, 14, 15, 28, 29], three (21.4%) had a moderate risk [30–32], and the remaining six studies (42.9%) had a high risk of bias [16, 17, 33–36].

**Table 2:** The results of the quality assessment using the QUIPS tool

	Bias of domains								
References	Study participation	Study attrition	Prognostic factor measurement	Outcome measurement	Study confounding	Statistical analysis and reporting	Score	Risk of bias	Quality
Cheung [30]	2	1	2	2	2	2	11	Moderate	Moderate
Cheung [14]	2	2	2	2	2	2	12	Low	High
He [15]	2	2	2	2	2	2	12	Low	High
He [33]	2	0	1	2	2	1	10	High	Low
Ohrt-Nissen [31]	2	1	2	2	2	2	11	Moderate	Moderate
Ohrt-Nissen [32]	2	2	2	2	2	1	11	Moderate	Moderate
Wong [28]	2	2	2	2	2	2	12	Low	High
Kuroki [34]	2	0	2	2	2	1	9	High	Low
Cheung [6]	2	2	2	2	2	2	12	Low	High
Kawasaki [36]	2	1	2	2	2	1	10	High	Low
Strube [17]	2	2	1	2	1	1	9	High	Low
Falbo [16]	2	2	1	2	2	1	10	High	Low
Kwan [29]	2	2	2	2	2	2	12	Low	High
Kuroki [35]	2	2	2	1	2	1	10	High	Low

*Study participation*: The key sample represents the population of interest on key characteristics, sufficient to limit potential bias to the results

*Study attrition*: Loss to follow-up (from sample to study population) is not associated with key characteristics, sufficient to limit potential bias (i.e., the study data adequately represent the sample)

*Prognostic factor measurement*: The prognostic factor of interest is adequately measured in study participants to sufficiently limit potential bias

*Outcome measurement*: The outcomes of interest are adequately measured in study participants to sufficiently limit potential bias

*Study confounding*: Important potential confounders are appropriately accounted for, limiting potential bias with respect to the prognostic factor of interest

*Statistical analysis and reporting*: The statistical analysis is appropriate for the design of the study, limiting potential for presentation of invalid results

The results of the GRADE rating are presented in Table 3. Most studies utilized a retrospective design. Therefore, the majority of methodological shortcomings of the included studies were related to loss of follow-up in domains of study attrition.

**Table 3:** The GRADE quality of evidence rating

Potential prognostic	NO	Studies	Design	Downgrading factors					Upgrading factors			
				ROB	Inconsistency	Indirectness	Imprecision	Publication bias	Moderate/Large effect size	Dose effect	Confounding	Overall quality
Flexibility	2261	14	Exploratory	Yes	Yes	No	No	No	Yes	No	Yes	Moderate

*ROB* Risk of bias, *NO.* Number of subjects

The results of interrater reliability for the risk of bias assessment between the reviewers indicated a high level of reliability, with an agreement rate of 96% and a kappa coefficient of 0.87.

### Study characteristics

The total sample size of AIS participants included in this review was 2261. Among the fourteen articles from eight research teams, eight were retrospective studies, and six were prospective. A summary of the study characteristics is shown in Table 4.

**Table 4:** Basic characteristics of the included studies

References	Country	Study type	Subjects, Age(mean years), Sex	Risser	Initial Cobb (°)	Curve type/pattern	Type of brace	Duration of brace	Follow-up duration	Outcomes	Definition of outcomes
Cheung [30]	HK	R	586AIS,12.6, 79M, 507F	0–2	30.9	249T, 337L	Underarm brace	NA	NA	Curve progression	The post-brace Cobb angle with > 5° increase from the pre-brace Cobb angle
Cheung [14]	HK	R	105AIS,12.2, 8M, 97F	0–3	31.7	72D, 33S	Underarm brace	NA	NA	In-brace Cobb	Cobb angle obtained from the in-brace radiograph when the patient wears the brace for 2 weeks
He [15]	HK	P	35AIS, 12, 3M, 32F	0–2	28	32D, 3S	HK orthoses	NA	NA	Initial in-brace correction	(Angle X-ray standing − Angle X-ray in-orthosis)/Angle X-ray standing
He [33]	HK	P	22AIS, 12, 2M, 20F	0–2	28.1	21D, 1S	HK orthoses	NA	NA	In-brace curvature	Curvature angle obtained from the in-brace radiographs and ultrasound imaging when the patient wears the brace for 2–3 weeks
Ohrt-Nissen [31]	Denmark	R	63AIS, 13.3, 3M, 60F	0–2	34	37T, 12TL, 5L, 9DM	Providence brace	26 months	2 years	Curve progression	Progression of ≥ 6°at skeletal maturity
Ohrt-Nissen [32]	Denmark	R	127AIS, 13.6, 14M, 113F	NA	35	67T, 27TL, 10L, 23DM	Providence brace	NA	NA	Initial in-brace Cobb	Cobb angle on initial in-brace radiograph
Wong [28]	HK	P	207AIS, 12.8, 35M, 172F	0–5	31.7	110T, 97L	Boston or Milwaukee bracing	NA	NA	Curve progression	An increase in major curve Cobb angle > 5° on the outcome radiograph compared to baseline, or the incidence of surgery
Kuroki [34]	Japan	R	31AIS, 12, 2M, 29F	0–2	27.3	4T, 4TL, 12L, 7DM, 1DT, 3TM	(OMC) brace	4 years and 8 months	During brace wear: 3 years and 4 months; Post-brace weaning: 1 year and 4 months	Curve progression	Increase of the Cobb angle by 6° or more, progression beyond the Cobb angle of 45° who were considered candidates for surgery
Cheung [6]	HK	R	586AIS, 12.6, 79M, 507F	0–2	31	251T, 335TL/L	Underarm TLSO brace	3.8 years	2.0 years	Curve regression; Curve progression	Curve regression: at least 5° reduction in the Cobb angle; Curve progression: at least 5° increase in the Cobb angle
Kawasaki [36]	Japan	R	133AIS, 12.2, 21M, 112F	0–2	31.9	62T, 28TL, 43 double/triple curves	Underarm TLSO brace	1.7 years	NA	Curve progression	Cobb angle > 6° identified from out-of-brace radiographs
Strube [17]	Germany	R	127AIS,13.1, 17M, 110F	0–2	28(median)	23D, 104S	Chêneau brace	2.1 years	NA	Failure and success	Failure: progression of curve to ≥ 45° or surgery needed during or after treatment or weaning up until the time of data acquisition; Success: progression of curve to < 45°, no surgery
Falbo [16]	USA	P	17AIS, 11.82, NA	0–3	27.63	14D, 3S	TLSO	NA	NA	In-brace correction	NA
Kwan [29]	HK	P	46AIS, 12.1, 4M, 42F	0–2	30	15T, 31TL	TLSO	NA	3.2 years	Curve progression	Cobb angle worsened by ≥ 6° or reached the threshold for surgical treatment at a minimum of 2 years of bracing or the time of the latest follow-up
Kuroki [35]	Japan	P	176AIS, 13.1, 14M, 162F	0–5	31	62T, 23TL, 22L, 14DT, 42DM, 13TM	OMC brace	NA	NA	Initial brace Cobb angle	NA

*R* Retrospective; *P* Prospective; *M* Male; *F* Female; *HK* Hong Kong; *OMC* Osaka Medical College; *T* Thoracic major curve; *L* Lumbar major curve; *TL* Thoracolumbar major curve; *D* Double curves; *S* Single curve; *DT* Double thoracic; *DM* Double major; *TM* Triple major; *NA* Not available

There was a wide range of sample sizes among the included studies (ranging from 17 to 586). Of all the samples, 1587 subjects were from China (all were from Hong Kong); 340 subjects were from Japan; 190 were from Denmark; 127 patients were from Germany; and 17 patients were recruited in the USA. The initial mean age of the patients was approximately 12–13 years. The range of the mean pre-brace Cobb angle was from 27.3° [34] to 35° [32]. However, the gender distribution information was missing in one study [16]. All studies presented the curve types or patterns.

For six studies conducted by three research teams, AIS patients were prescribed an underarm thoraco-lumbo-sacral orthosis (TLSO) [6, 14, 16, 29, 30, 36]. In another two studies from one team, a Hong Kong orthosis was prescribed [15, 33]. The Providence nighttime brace was used in two other studies [31, 32]. The Chêneau brace was used in one study [17], and the Osaka Medical College (OMC) brace was prescribed in two studies [34, 35]. In addition, one study used the Boston or Milwaukee brace [28].

In these studies, the bracing outcomes were defined with four indicators, including the initial in-brace Cobb angle [14, 32, 33, 35], initial in-brace correction rate [15, 16], curve progression [17, 28–31, 34, 36], and curve regression [6]. For the initial in-brace correction rate, six studies calculated with major curve magnitudes [6, 28, 30–32, 36], four studies considered both major and minor curves [14, 15, 17, 33], and the remaining four studies had no clarification [16, 29, 34, 35].

### Evidence synthesis

All the included studies provided information about the measurement methods of spinal flexibility and reported the predictive effect of flexibility for bracing outcomes. The detailed results related to the two above-mentioned review objectives are described separately below.

#### Measurement methods of spinal flexibility

A total of nine flexibility assessment methods were identified in the included 14 articles, and the radiographic method with different postures was the most common method, which was used in 12 studies. Seven papers [6, 14, 28–30, 33, 36] reported that supine radiographs could be used to measure spinal flexibility with the formulation of (pre-brace Cobb angle—supine Cobb angle)/pre-brace Cobb angle * 100%. Two papers published by one research center described the method of supine lateral bending radiographs [31, 32]. Lateral bending radiographs with unclear positions were used in one paper [17]. Kuroki et al. provided a novel method with a hanging spine X-ray to present spinal flexibility [34, 35]. Falbo et al. documented the flexibility of the spine with the physical fulcrum bending method and recorded the value of flexibility based on a visual guide [16].

Ultrasound imaging could also be an alternate method for measuring spinal flexibility before bracing for patients with AIS. In the two studies conducted by He et al., the ultrasound images of the full spine in the supine, prone, seated with side bending, and prone with side bending positions were captured by an ultrasound device called “Scolioscan” and compared with the upright images to assess the flexibility [15, 33]. The flexibility was defined as the ratio calculated by the curve angle in upright ultrasound images deducting the curve angle in the given positions and then dividing the curve angle in upright images. A summary of the measurement methods is shown in Table 5.

**Table 5:** Summary of spinal flexibility assessment methods

References	Methods	Positions	Parameters	Definitions
Cheung [14]	Radiographic	Supine	Supine Cobb angle	NA
Cheung [30]	Radiographic	Supine	Flexibility rate	(Pre-brace Cobb angle − supine Cobb angle)/pre-brace Cobb angle × 100%
Cheung [6]	Radiographic	Supine	Curve flexibility	(Pre-brace Cobb angle − supine Cobb angle)/pre-brace Cobb angle × 100%
He [15]	Radiographic and ultrasound	Supine Prone Prone with lateral bending Sitting with lateral bending	Curve flexibility	(Angle US _standing_ − Angle US _in given position_)/Angle US _standing_
He [33]	Radiographic and ultrasound	Supine Prone Prone with lateral bending Sitting with lateral bending	Curvature angle in four positions	NA
Ohrt-Nissen [31]	Radiographic	Supine lateral bending	Curve flexibility	(Standing Cobb angle − Supine lateral bending Cobb angle)/Standing Cobb angle × 100%
Ohrt-Nissen [32]	Radiographic	Supine lateral bending	Curve flexibility	(Standing Cobb angle − Supine lateral bending Cobb angle)/standing Cobb angle × 100%
Wong [28]	Radiographic	Supine	Supine flexibility rate	(Pre-brace Cobb angle − supine Cobb angle)/pre-brace Cobb angle × 100%
Kuroki [34]	Radiographic	Hanging	Flexibility index	(Cobb angle in upright position − Cobb angle in hanging position)/Cobb angle in upright position × 100%
Kawasaki [36]	Radiographic	Supine	Supine flexibility rate	(Standing Cobb angle − initial supine Cobb angle)/standing Cobb angle × 100%
Strube [17]	Radiographic	Bending toward the convexity	Curve flexibility	Change in Cobb angle between AP view and bending to the convex side (deltaCobb1_bend_/deltaCobb2_bend_)
Falbo [16]	Physical	Fulcrum bending	Curve flexibility	A visual recording method
Kwan [29]	Radiographic	Supine	Supine flexibility	(Pre-brace Cobb Angle − Supine Cobb Angle)/Pre-brace Cobb Angle × 100%
Kuroki [35]	Radiographic	Hanging	Flexibility index	(Cobb angle in upright position − Cobb angle in hanging position)/Cobb angle in upright position × 100%

*NA* Not available; *US* Ultrasound; *AP* Anteroposterior

#### Associations of spinal flexibility with the treatment outcomes of brace

A summary of the association between spinal flexibility and brace correction outcomes is presented in Table 6. Of the fourteen studies, only five papers reported the correlation coefficient of flexibility with the initial in-brace Cobb or correlation rate. Cheung et al. found a significant correlation (*r* = 0.65 [30], *r* = 0.74 [14]) between the supine flexibility rate measured from radiographs and the immediate in-brace correction rate. A regression model was developed in one of their studies, which generated a regression of 0.809 between the in-brace Cobb angle and the supine Cobb angle [14]. Kuroki et al. concluded that there was a significant correlation (*r* = 0.762) between the Cobb angle measured in hanging spine X-rays and that measured in initial in-brace radiographs [35]. He et al. conducted a prospective study and demonstrated that spinal flexibility measured by ultrasound imaging in the prone position was significantly correlated with in-brace correction (*r* = 0.75 [15], *r* = 0.87 [33]). Moreover, six studies were identified with predictive models to investigate the relationship between spinal flexibility and bracing outcomes [6, 28–31, 36]. All of the prognostic studies used a multivariate logistic regression model to study different predictive factors. Spinal flexibility was determined as one of the significant predictors in each study. The summary of the six prognostic studies is shown in Table 7.

**Table 6:** Associations between the spinal flexibility and bracing outcomes

References	Pre-brace flexibility (mean value)	Post-brace correction (mean value)	Correlation	Primary outcomes	Findings
Cheung [30]	Supine flexibility rate (30.20%)	First in-brace correction rate (41%)	*r* = 0.650; *p* < 0.001	Curve progression	Supine flexibility can predict in-brace correction and risk of curve progression
Cheung [14]	Supine Cobb angle/supine flexibility rate (22.5°/70.6%)	Immediate in-brace Cobb angle/in-brace correction rate (18.9°/85.1%)	*r* = 0.740; *p* < 0.001	Immediate in-brace Cobb	Supine radiographs have predictive value for in-brace correction of AIS, the in-brace Cobb angle is 0.809 of the supine Cobb angle
He [15]	Supine flexibility (40%)	Initial in-brace correction (41%)	*r* = 0.660; *p* > 0.05	Initial in-brace correction	The spinal flexibility in the prone position is the closest to and most correlated with the initial in-orthosis correction among the four studied positions. The prone position could be an effective method to predict the initial effect of orthotic treatment on the patients with AIS
	Prone flexibility (42%)		*r* = 0.750; *p* > 0.05		
	Flexibility of sitting with lateral bending (143%)		*r* = 0.040; *p* < 0.05		
	Flexibility of prone with lateral bending (127%)		*r* = 0.030; *p* < 0.05		
He [33]	Supine Cobb angle (radiograph) (18.8°)	In-brace Cobb (16.6°)	*r* = 0.730; *p* < 0.05	In-brace Cobb	The recumbent curvatures (especially prone curvature) could be a predictor of the initial effect of orthotic treatment in the patients with AIS
	Supine curvature (ultrasound) (10.7°)	In-brace curvature (11.2°)	*r* = 0.760; *p* = 0.27	In-brace curvature	
	Prone curvature (ultrasound) (10.7°)		*r* = 0.870; *p* = 0.16		
	Curvature of sitting with lateral bending (ultrasound) ( − 6.5°)		*r* < 0.30; *p* < 0.05		
	Curvature of prone with lateral bending (ultrasound) ( − 3.5°)		*r* < 0.30; *p* < 0.05		
Ohrt-Nissen [31]	Supine bending curve flexibility rate (60%)	Immediate in-brace correction (61%)	NA	Curve progression	A decrease in curve flexibility, as determined by supine lateral bending radiograph, was an independent predictor of curve progression
Ohrt-Nissen [32]	Supine bending curve flexibility rate (63%)	Initial in-brace correction (63%)	NA	Initial in-brace Cobb	Supine lateral bending radiographs may serve as a key prognostic parameter in patients with AIS before initiating brace treatment
Wong [28]	Supine flexibility rate (23.2%)	First in-brace correction rate (33.7%)	NA	Curve progression	Flexibility was found to be significantly predictive of curve for curve progression; A higher supine flexibility (18.1%) predicted a lower risk of progression
Kuroki [34]	Flexibility index (NA)	Initial correction rate (NA)	NA	Curve progression	Curve flexibility did not affect the clinical results of brace treatment. However, success rate was insignificantly higher in the cases whose Cobb angle in brace was smaller than that in hanging position
Cheung [6]	Supine flexibility rate (30%)	First in-brace correction rate (41%)	NA	Curve regression; Curve progression	Despite a trend for patients with curve regression to have higher baseline flexibility, after controlling for other factors, no clinically important differences was found with increased flexibility
Kawasaki [36]	Supine flexibility rate (22%)	Initial in-brace correction rate (31.7%)	NA	Curve progression	Those with higher flexibility are at risk of curve progression
Strube [17]	Curve flexibility (NA)	In-brace correction (NA)	NA	Curve progression	Treatment failure depended significantly on major curve flexibility (*p* = 0.005)
Falbo [16]	Curve flexibility (59.64%)	In-brace correction (23.57%)	NA	In-brace curve correction	Curve flexibility alone cannot predict coronal curve correction. Additional factors must be considered when predicting success of brace treatment for AIS
Kwan [29]	Supine flexibility (43.9%)	Immediate in-brace correction rate (47.9%)	NA	Curve progression	Curve flexibility was associated with an increased risk of curve progression
Kuroki [35]	Hanging Cobb angle (21.1°)	Initial in-brace Cobb (20.3°)	*r* = 0.762; *p* < 0.001	Initial brace Cobb angle	Hanging total spine X-ray is useful for confirmation of adequate correction by the OMC brace in idiopathic scoliosis

OR Odds ratio; *CI* Confidence interval; *NA* Not available; *OMC* Osaka Medical College

**Table 7:** Summary of predictive model for the bracing outcomes

References	Significant factors	Outcome indicators	Predictive model
Cheung [6]	Age (*p* = 0.01) Pre-menarche at baseline (*p* = 0.01) Correction rate (*p* = 0.04) Flexibility (*p* = 0.03) Change in the apical ratio (*p* < 0.01)	Curve progression	Multivariate logistic regression model
Wong [28]	Sacral slope (*p* = 0.002) Pelvic incidence (*p* = 0.005) Flexibility (*p* < 0.001) Correction rate (*p* < 0.001)	Curve progression	Multivariate logistic regression model
Kwan [29]	Curve flexibility (*p* = 0.042) Immediate in-brace correction rate (*p* = 0.019) Pre-brace AVR (*p* = 0.049) AVR correction velocity at 1 year (*p* = 0.026)	Curve progression	Logistic regression analysis
Cheung [30]	Age (*p* < 0.001) Risser stage (*p* < 0.001) Curve type (thoracic vs lumbar) (*p* = 0.022) Pre-brace Cobb angle (*p* = 0.020) Correction rate (*p* = 0.001) Flexibility rate (*p* < 0.001)	Curve progression	Multivariate logistic regression model
Ohrt-Nissen [31]	Flexibility (*p* = 0.013) Premenarchal status (*p* = 0.002)	Curve progression	Multivariate logistic regression model
Kawasaki [36]	Flexibility rate (*p* = 0.045) Correction rate (*p* = 0.034) Risser sign (*p* = 0.032)	Curve progression	Multivariate logistic regression model

A total of eleven papers illustrated that spinal flexibility could be a predictive factor of the bracing outcome, which consisted of four high-quality studies [14, 15, 28, 29], three moderate-quality studies [30–32], and four low-quality studies [17, 33, 35, 36]. Cheung et al. reported a significant association of curve progression with flexibility rate (OR = 0.958, 95% CI = 0.943–0.974) and found a flexibility cutoff value of 0.28 for curve progression under the receiver operating characteristic curve [30], while Wong et al. demonstrated that patients with higher flexibility could predict a lower possibility for progression (OR = 0.947, 95% CI = 0.910–0.984) and identified a cutoff value of 0.181 for flexibility in predicting curve deterioration [28]. Ohrt-Nissen et al. supported that a one-percent increase in flexibility could be significantly associated with a decreased risk of curve progression by more than 5° (OR = 0.950, 95% CI = 0.900–0.980) [31]. However, three other papers reported no significant relationship between spinal flexibility and the clinical results of brace treatment [6, 16, 34].

There were some conflicting results in some studies, even with the same authors. Two retrospective studies conducted by Cheung’s team indicated that the flexibility measured with supine radiographs could provide a satisfactory prediction for determining the brace effect [14, 30]. However, their other studies demonstrated that although patients with curve regression have a tendency to have greater baseline flexibility, after controlling for the patient’s age, Risser sign, Sanders stage, and radius and ulnar grade, no clinically significant differences were found between the curve regression and the increased flexibility (OR = 1.010, 95% CI = 0.980–1.030, *p* = 0.69) [6]. Similarly, Kuroki et al. evaluated spinal flexibility by hanging a total spine X-ray prior to OMC brace intervention and reported that hanging flexibility is beneficial for confirming adequate correction of the OMC brace [35]. Nevertheless, they also explored the predictive factors of the OMC brace in another study and found that spinal flexibility did not influence the clinical outcomes of brace treatment [34].

Among the fourteen included studies, curve types or patterns of the patients were shown as the basic characteristics. However, only eight studies mentioned the relationship between curve patterns and spinal flexibility or bracing outcomes [6, 13–15, 30, 32–34]. Kuroki et al. [33], Falbo et al. [14], and He et al. [13] found that the association between spinal flexibility and correction results was independent of curve patterns. Strobe et al. [15] reported that the success rate was higher for a single lumbar curve than for a thoracic curve. On the other hand, Kuroki et al. [32] claimed that the success rate of thoracolumbar curves tended to be higher than that of thoracic and lumbar curves. Although these studies discussed the impact of curve types on the treatment success rate, they did not analyze the relationship between pre-brace flexibility and bracing outcomes in different curve patterns.

## Discussion

This literature review provides information regarding the assessment methods and the prediction of spinal flexibility prior to brace treatment in AIS. Nine methods were identified for measuring spinal flexibility in bracing candidates with AIS, and the radiographic method was the most compelling. Moderate evidence supported spinal flexibility as a predictive factor for bracing treatment outcomes.

This review summarized the evaluation methods and found that radiographic assessment was the most widely used. Nevertheless, considering the radiative effect of radiography, reducing the additional X-ray ionizing radiation is the primary consideration for clinicians and patients when assessing spinal flexibility. Chevrefils et al. proposed a novel flexibility assessment method through MRI texture analysis [20], but its application on brace candidates with AIS has not been explored previously. In addition, He et al. estimated the curve correction in braces with the ultrasound system and confirmed the feasibility of flexibility measurement with ultrasound imaging in the prone position [15]. Their findings corresponded to another study [37], in which the reliability of the spinal flexibility assessment using ultrasound technique on nonoperative candidates with AIS was determined. Therefore, ultrasound imaging has been found to be a potential radiation-free method for assessing spinal flexibility before bracing treatment and should be promoted in future research.

In terms of the positions for flexibility assessment, among the nine methods of flexibility assessment, the supine, prone, lateral bending, and fulcrum bending positions were identified, but there was a lack of comparison between different positions of spinal flexibility measurement for patients who received brace treatment. It is still inconclusive which position could better predict bracing treatment outcomes. Therefore, more comparative research on different positions and techniques of flexibility assessment should be conducted.

Moreover, the type of brace may influence the recognition of predictive factors for bracing outcomes in AIS. Moradi et al. conducted a systematic review to identify the clinical and radiological factors for predicting outcomes of overcorrection nighttime bracing and found that better curve flexibility and a higher Risser stage were significantly correlated with the success of overcorrection nighttime bracing [38]. Bogaart et al. evaluated predictive factors for the correction outcome of the TLSO brace, and their results suggested that insufficient initial in-brace correction had a strong correlation with bracing failure, while curve flexibility had no relationship with treatment success or failure [9]. Our study included both the Providence nighttime brace and the TLSO, but the results showed that spinal flexibility can predict curve progression for bracing candidates.

The curve pattern is an influential factor of bracing outcomes. Thompson et al. concluded that the thoracic curves have a greater risk for brace failure than the lumbar curves [39]. They claimed that the change in curve pattern may imply flexibility and is associated with brace success. This review included fourteen studies regarding the relationship of spinal flexibility with brace treatment outcomes but without detailed analysis concerning the effect of spinal flexibility on bracing outcomes in different curve patterns. Ohrt-Nissen et al. [30] found significantly less correction in thoracic curves than in other curve types, but when curve correction was adjusted for curve flexibility, there was no statistical difference between curve types. Kawasaki et al. [34] reported that thoracolumbar or lumbar curves have higher correction rates and flexibility rates than thoracic and double or triple curves. In contrast to these findings, Strobe et al.’s study demonstrated better outcomes for double curves in scoliosis with a thoracic major curve [15]. With the influence of the rib cage, patients with thoracic major curves may suffer lower curve correction rates due to the less flexible structure. However, higher flexibility rates for thoracic curves may also be more likely for out-of-brace curve progression [34]. As insufficient evidence about curve pattern effects with spinal flexibility in bracing outcomes, future research is encouraged to provide a deeper understanding of this specific area of interest.

Flexibility in this review refers mainly to the ability to decrease the curvature when gravity is eliminated or applied forces are changed in different situations. Hippocrates first proposed the essence of curve flexibility evaluated with the use of physical forces. Traction and gravitational forces were applied to enable the inherent flexibility of the curve to emerge and facilitate the curvature correction [40]. Namely, the flexibility actually describes the correctability of the spinal deformity and inherently represents the ability of the spine curve to change under external forces [41]. Brace types may affect the predictive value of flexibility as the applied force of different braces varies. A similar flexibility rate before bracing treatment may suffer significant differences among the results of a very rigid brace, a rigid brace, and an elastic brace. Accordingly, a clear description of the brace classification in line with the study of Negrini et al. might be helpful for further determining the predictive ability of spinal flexibility [42].

In this study, curve progression was defined as the treatment outcome in seven articles with different criteria, and several studies regarded the post-brace Cobb angle with a 5° or more increase compared to the baseline as progression [6, 28, 30]. Ohrt-Nissen et al. and Kawasaki et al. defined curve progression as an increase of more than 6° [31, 36]. Similarly, two other studies also considered a Cobb angle worsened by more than 6° as the curve progressed, but progression to the surgical threshold was also one of the criteria [29, 34]. Therefore, the inconsistent outcome measures and definitions in these studies might limit the general application of the findings in this review. The Scoliosis Research Society (SRS) established research criteria for AIS bracing studies in 2005. According to SRS criteria, treatment outcomes of the brace should be presented as the percentage of patients who have 6° or more progression at skeletal maturity or progress beyond 45° to the possible need for surgery [43]. A consensus reached in 2014 by SRS and SOSORT also recommended clearly delineated outcome measures [44]. Therefore, adherence to these criteria and recommendations would facilitate the interpretation of future clinical studies.

Furthermore, the success or failure of bracing is not solely dependent on spinal flexibility; other influential factors, such as skeletal maturity, curve patterns, and curve location, should be taken into account accordingly [45, 46]. In addition, research has found that elastic scapular taping [47], myofascial release [48], and exercises [49–52] could improve flexibility for patients with scoliosis, so adopting suitable strategies to improve spinal flexibility, thereby improving the corrective effect of bracing, should be further explored.

### Limitations

This study has several limitations. First, the language filter applied in the retrieval strategies in this review may have led to a narrow number of included studies and thus affected the main findings. Second, the OR of spinal flexibility as a risk factor for bracing treatment failure and the correlation coefficient of flexibility with bracing outcomes, such as the initial in-brace correction rate, which were the main factors to consider in this systematic review, were not provided in all included studies, and several studies did not directly report the correlation between flexibility and post-brace outcomes. The results were then analyzed and computed based on the reported information, which may have contained human errors and bias. Finally, there were some vague descriptions of the statistical analysis methods and reported results. Future studies in this direction should minimize the inconsistency and report complete information related to the methods and findings.

## Conclusion

This review comprehensively analyzed the evaluation methods and predictive value of spinal flexibility prior to brace treatment for the first time and identified nine measurement methods of spinal flexibility for bracing candidates with AIS. Among them, the supine radiograph was the most commonly used method, and ultrasound in the prone position was a promising non-radiative choice before bracing. In addition, pre-brace flexibility was strongly correlated with the in-brace Cobb angle or correction rate, and moderate evidence supported that spinal flexibility could predictively determine the treatment outcomes of brace.

## Data Availability

The datasets generated during and/or analyzed during the current study are available throughout the manuscript.
